# WormRuler: A software to track body length used to characterize a super red-shifted channelrhodopsin in *Caenorhabditis elegans*

**DOI:** 10.17912/micropub.biology.000607

**Published:** 2022-07-19

**Authors:** Marius Seidenthal, Dennis Vettkötter, Alexander Gottschalk

**Affiliations:** 1 Buchmann Institute for Molecular Life Sciences, Goethe-University, Max-von-Laue-Strasse 15, D-60438 Frankfurt, Germany; 2 Department of Biochemistry, Chemistry, and Pharmacy, Institute for Biophysical Chemistry, Goethe-University, Max-von-Laue-Strasse 9, D-60438 Frankfurt, Germany

## Abstract

Manipulation of neuronal or muscular activity by optogenetics or other stimuli can be directly linked to the analysis of *Caenorhabditis elegans* ( *C. elegans* ) body length. Thus, WormRuler was developed as an open-source video analysis toolbox that offers video processing and data analysis in one application. Utilizing this novel tool, the super red-shifted channelrhodopsin variant, ChrimsonSA, was characterized in *C. elegans* . Expression and activation of ChrimsonSA in GABAergic motor neurons results in their depolarization and therefore elongation of body length, the extent of which providing information about the strength of neuronal transmission.

**Figure 1. WormRuler software can be used to identify changes in body length in C. elegans .**
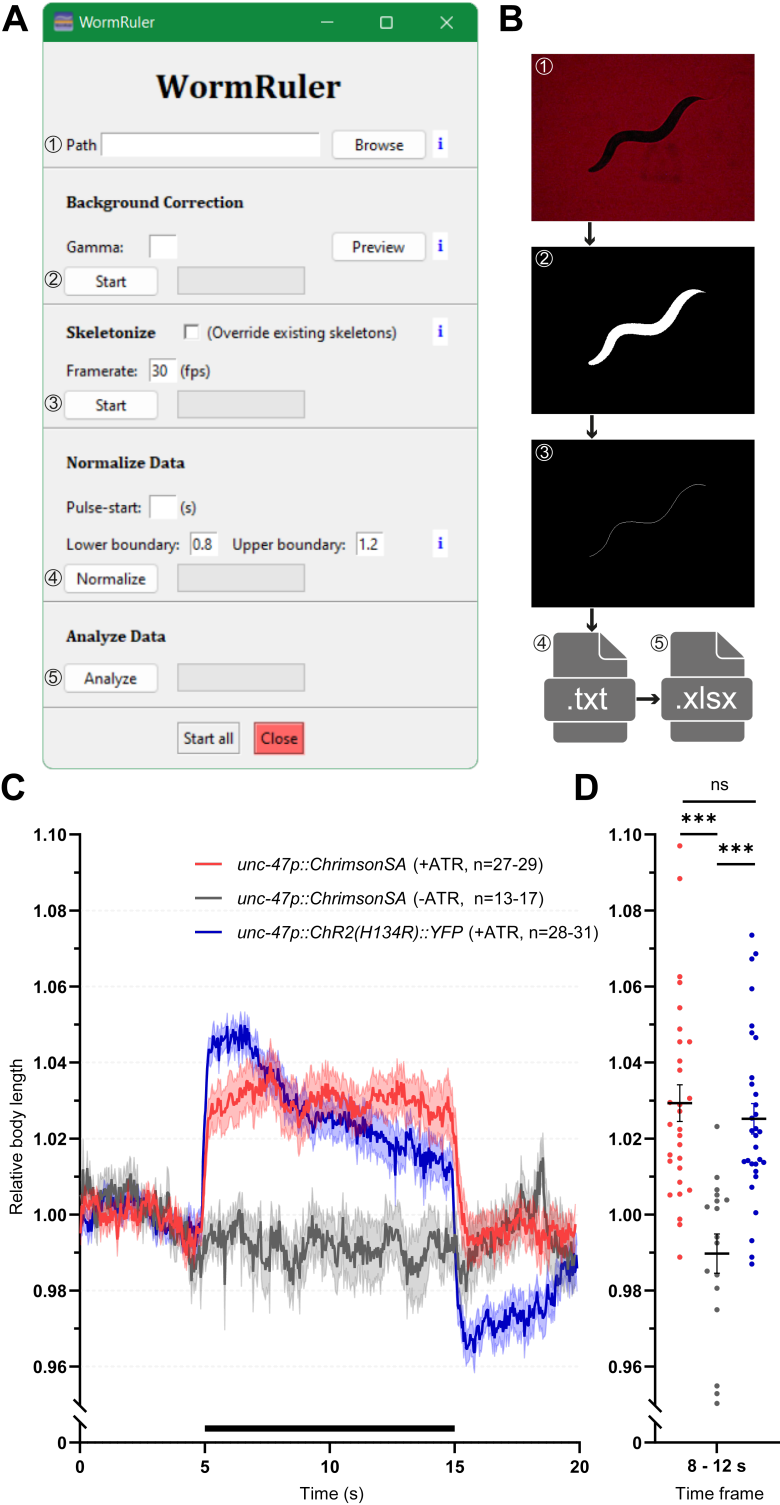
**A)** The graphical user interface (GUI) of WormRuler. **B)** The input and outputs of WormRuler. (1) An example still image of a raw video recorded of a single worm. Example images of the output videos after background correction (2) and skeletonization (3). Processing steps “normalize data” (4) and “analyze data” (5) result in *.txt and *.xlsx formats, respectively. **C)** Mean (+SEM) relative body length of animals expressing *unc-47p::ChrimsonSA* or *unc-47p::ChR2(H134R)::YFP* . Videos of animals with (+) or without (-) ATR were acquired and analyzed with WormRuler. A 10 s light stimulus (either 590-650 nm for ChrimsonSA activation or 450-490 nm for ChR2(H134R) activation) was applied after 5 s. Experiments were performed on 2-3 days with animals picked from different plates. **D)** Group analysis of data in (C), interval during (8-12 s) illumination with n=29 (ChrimsonSA, +ATR), n=17 (ChrimsonSA, -ATR) and n=31 (ChR2, +ATR). Unpaired t-test; *p<0.05, **p<0.01, ***p<0.001, ns not significant; error bars are s.e.m; n is the total number of animals.

## Description

Measurement of body length during optogenetic stimulation may be used to investigate defective neuromuscular transmission or altered muscular function in *Caenorhabditis elegans* and other model organisms (Nagel *et al.* , 2005; Liewald *et al.* , 2008; Meloni *et al.* , 2020). The WormRuler tool has been developed as an open-source Python-based platform to detect changes in body length during stimulation (Fig. 1A) (Van Rossum & Drake Jr, 1995). To this end, videos of single freely moving animals are analyzed (Fig. 1B). As the first step, WormRuler performs a background correction to detect the outlines of the animal by creating a binarized version of the video (Fig.1B) (van der Walt *et al.* , 2014). Differences in backlight intensity may be compensated by adjusting the “Gamma” value which can be tested by using the preview button (Fig. 1A). By the selection of a ROI, rims or structures recorded around the animal can be avoided. Subsequently, the binary version will be thinned to a skeleton (van der Walt *et al.* , 2014) and further processed into filamentary structures (Fig. 1B) (Koch & Rosolowsky, 2015). As the resulting structures can contain many branches, an additional analysis is performed to identify the length of the longest path through the skeleton (Koch & Rosolowsky, 2015). To be able to compare different animals, the length of the skeleton given in pixels is normalized to the average length from time points before the presented stimulation light pulse. Relative body length values below 0.8 and 1.2 are automatically discarded as they reflect unwanted effects such as coiling or artifacts (Tolstenkov *et al.* , 2018). These boundaries can be adjusted if wanted. In the final step, the normalized data, as well as calculations of mean values (including standard error of the mean and the number of analyzed animals), are exported to an excel file for subsequent plotting of data and statistical analysis. When more than 50 % of the datapoints of an animal are omitted, it will be ignored in the statistical analysis and listed separately. Files reporting each step of the analysis are generated so the researcher may confirm the produced results.

For validation of its applicability, WormRuler was used to establish the recently described super red-shifted channelrhodopsin variant ChrimsonSA as a novel optogenetic tool for depolarization of *C. elegans* motor neurons (MNs) (Oda *et al.* , 2018). Optogenetic activation of GABAergic MNs leads to relaxation of body wall muscles and thus elongation of the animal (Liewald *et al.* , 2008). By using the promoter of *unc-47* , ChrimsonSA will be expressed in GABAergic MNs and thus an increase in body length upon illumination with 620 nm light can be observed (McIntire *et al.* , 1997; Liewald *et al.* , 2008). By comparing animals treated with and without the chromophore *all-trans-retinal* (ATR), we could show that animals expressing ChrimsonSA + ATR show a significant increase in body length during a 10 s light stimulus, whereas worms that lack ATR display no elongation (Fig. 1C+D). In comparison, expression of the channelrhodopsin‑2 (ChR2) gain-of-function mutant H134R in GABAergic neurons results in about 4 % of elongation, compared to about 3 % in ChrimsonSA expressing animals (Fig. 1C, Liewald *et al.* , 2008). However, in contrast to ChR2, ChrimsonSA expressing animals do not show a decline of body length during stimulation. Thus, the ChrimsonSA variant is functional and may be used to depolarize MNs in *C. elegans* making it a useful addition to the optogenetic tool box, particularly in combination with blue-light activated tools or reporters.

In sum, WormRuler is a simple, free-to-use tool with a user-friendly interface for the analysis of the body length of *C. elegans,* either for elongation, as shown in this study, or for contraction (see example video in GitHub repository). While WormRuler was developed to study *C. elegans* , we suggest that it can be a useful tool to characterize the length of other model organisms with an elongated body posture (e.g. *Drosophila melanogaster* larvae).

## Methods

Animals were kept at 20°C on nematode growth medium (NGM) plates containing OP50 (Brenner, 1974) optionally containing 200 µM ATR. Transgenic L4 larvae were placed onto ATR plates about 18 h before the experiment. The acquisition was performed on empty NGM plates using an Axio Scope A1 (Zeiss) microscope equipped with a 10x objective and a Powershot G9 digital camera (Canon, 30 fps, 640 x 480 pixels). Animals were tracked manually by moving the microscope stage and stimulated with a 10 s light pulse (1 mW/mm ^2^ , 590-650 nm for ChrimsonSA activation or 450-490 nm for ChR2(H134R) activation) from a 50-W HBO lamp. Brightfield light was filtered (665-715 nm) to avoid unwanted activation of the respective channelrhodopsin. The duration of the light stimulation was regulated by a computer-controlled shutter (Lambda SC SmartShutter, Sutter Instruments). Video acquisition and shutter opening were controlled through an Arduino UNO device (Arduino) running a custom-written script. Subsequently, videos were processed with WormRuler (available from https://github.com/dvettkoe/wormruler ). GraphPad Prism 8 was used to plot the data and to perform statistical tests.

## Reagents

**Table:** 

Strain	Genotype	Source
ZX3081	*zxEx1361[100 ng/µl unc-47p::ChrimsonSA; 1.5 ng/µl myo-2p::mCherry]*	This study
ZX426	*zxIs3[80 ng/µl unc-47p::ChR2(H134R)::YFP]*	Liewald et al. 2008

## Extended Data

Description: WormRuler Software. Resource Type: Software. DOI: 10.22002/D1.20232

Description: Original videos and WormRuler analysis of the animals which are depicted in figure 1. Resource Type: Dataset. DOI: 10.22002/D1.20233
